# Preparation and Lithium-Ion Capacitance Performance of Nitrogen and Sulfur Co-Doped Carbon Nanosheets with Limited Space via the Vermiculite Template Method

**DOI:** 10.3390/molecules29020536

**Published:** 2024-01-22

**Authors:** Fang Yang, Pingzheng Jiang, Qiqi Wu, Wei Dong, Minghu Xue, Qiao Zhang

**Affiliations:** 1 College of Material Science and Engineering, Liaoning Technical University, Fuxin 123000, China; 18378093525@163.com (P.J.); 18378093525@139.com (Q.W.); 2 Jiangsu Jiaming Carbon New Material Co., Ltd., Lianyungang 222300, China; xueminghu1213@163.com (M.X.); zhangqiao1213@outlook.com (Q.Z.)

**Keywords:** methylene blue, vermiculite, lithium-ion capacitor, carbon nanosheets

## Abstract

Nitrogen and sulfur co-doped graphene-like carbon nanosheets (CNSs) with a two-dimensional structure are prepared by using methylene blue as a carbon source and expanded vermiculite as a template. After static negative pressure adsorption, high-temperature calcination, and etching in a vacuum oven, they are embedded in the limited space of the vermiculite template. The addition of an appropriate number of mixed elements can improve the performance of a battery. Via scanning electron microscopy, it is found that the prepared nitrogen–sulfur-co-doped carbon nanosheets exhibit a thin yarn shape. The XPS results show that there are four elements of C, N, O, and S in the carbon materials (CNS-600, CNS-700, CNS-800, CNS-900) prepared at different temperatures, and the N atom content shows a gradually decreasing trend. It is mainly doped into a graphene-like network in four ways (graphite nitrogen, pyridine nitrogen, pyrrole nitrogen, and pyridine nitrogen oxide), while the S element shows an increasing trend, mainly in the form of thiophene S and sulfur, which is covalently linked to oxygen. The results show that CNS-700 has a discharge-specific capacity of 460 mAh/g at a current density of 0.1 A/g, and it can still maintain a specific capacity of 200 mAh/g at a current density of 2 A/g. The assembled lithium-ion capacitor has excellent energy density and power density, with a maximum power density of 20,000 W/kg.

## 1. Introduction

Nowadays, with the rapid development of society, higher requirements are put forward for the development of new energy conversion and storage techniques. In the context of the depletion of traditional energy and the destruction of the environment, it is very important to explore new clean and sustainable energy sources [1]. High energy density is the key point of battery development [2]. Based on the intermittence and randomness of new energy sources, exploring efficient and fast power storage devices will be the focus of future research.

Lithium-ion batteries (LIBs) [3] and supercapacitors (SCs) [4] are currently recognized as two promising energy storage devices. LIBs usually have high energy density, high operating voltage, and no memory effect. The main limitations of LIBs are low power density and poor cycle performance (within 4000 cycles). In contrast, SCs have advantages in power density, cycle life (usually more than 100,000 cycles), and safety but are limited by lower energy density (only 1/10 of LIB). In the practical application of vehicle energy storage systems and grid energy storage systems, it is usually necessary to have high energy density, high power density, and a long cycle life at the same time. Based on this, hybrid lithium-ion capacitors have begun to enter people’s vision, with a view to further improve energy density while maintaining high power density. Lithium-ion capacitors have higher energy density than conventional supercapacitors, which is mainly attributed to the high capacity provided by the lithium-ion insertion/extraction reaction of the anode material and the higher operating voltage generated by the lower anode lithium insertion potential [5]. The electrode potential of carbon materials is close to that of lithium metal, which has been widely used in lithium-ion batteries. Therefore, carbon-based materials are also the first choice for lithium-ion capacitor anode materials. However, how to develop low-cost and high-efficiency anode materials has become a huge challenge to achieving this goal.

Among various carbon-based materials, carbon nanosheets (CNSs) have been proven to be an advanced carbon-based material because of their large surface area and active reaction sites [6]. At the same time, their two-dimensional nanosheets are stacked to form a porous structure, which is very conducive to the adsorption and transportation of lithium ions [7]. The hard template method is one of the most commonly used methods for preparing two-dimensional carbon nanosheets, which can maintain the morphology of the precursor. They have the practicability of controllable morphology and an adjustable porous structure size that is easy to expand [8,9]. At present, many hard templates have been used to construct the two-dimensional structures of CNSs, such as nanosheets prepared using layered zinc hydroxide [10], porous MgO [11], NaCl salt [12], Na_2_SO_4_ salt [13], and g-C_3_N_4_ [14] or graphene oxide [15] as templates. However, most of these templates are artificially synthesized. The synthesis process is complicated and costly, which greatly limits the large-scale production and application of carbon nanotubes.

Natural vermiculite (VMT) is a silicate clay mineral formed because of turbidity or the weathering of mica. Its surface is generally a smooth-sheet, layered structure. VMT is a two-dimensional layered structure composed of layers (silicon–oxygen tetrahedron, magnesium–oxygen octahedron, etc.) and interlayer cations (hydroxyl molecules). Generally, VMT is easy to expand by more than 20 times through heat treatments, microwave irradiation, hydrogen peroxide expansion, and so on. The layered structure of VMT is used as a surfactant [16,17], which is widely used in the fields of green wastewater treatment, heat insulation, environmental protection planting, and electrocatalysis. The expanded vermiculite also shows excellent application potential in various fields. Expanded vermiculite is widely used as a 2D nanomaterial in the field of environmental adsorption by providing a nano-sized multilayer structure [18,19]. Liu et al. [20] first prepared chitosan–polyethylene–vermiculite hydrogel via the organic modification of vermiculite to achieve the efficient adsorption of methylene blue pollutants in an aqueous solution. In the field of electrochemical energy storage, Ning et al. [21] used expanded vermiculite as a template and ethylene as a carbon source to prepare ultra-large graphene nanosheets as electrode materials via chemical vapor deposition. However, the chemical vapor deposition method has the problem of a high preparation cost. Therefore, it is necessary to discuss a lower-cost way to study the application of vermiculite in the field of electrochemical energy storage.

The specific surface area and the number of micropores in the expanded vermiculite hard template increase, and such a template will be more conducive to the penetration of the electrolyte and provide more lithium-ion channels. By introducing a carbon layer, the conductivity of the electrode material is improved, and electron transport is accelerated. On the other hand, the excellent thermal expansion structure and chemically inert siloxane surface of vermiculite make it an ideal template for the in situ conversion of carbon-rich organic compounds into two-dimensional carbon nanomaterials. Heteroatom doping is also considered to be an effective strategy to improve the electrochemical performance of carbonaceous materials [22,23]. The two-dimensional network of N, S co-doped nanosheets can provide sufficient surface-active sites for Li^+^ adsorption and increase the interlayer spacing of Li^+^. The electronic conductivity, capacity, and rate performance of the composites are significantly improved, promoting electrode reaction kinetics. Biomass-derived carbons with elaborately designed nano-architectures can be achieved via activation or templating methods, in which defects, vacancies, and heteroatoms (such as N, S et al.) can also emerge in the pyrolysis procedure [24,25]. In this study, nitrogen and sulfur co-doped two-dimensional carbon nanosheet hard carbon materials were obtained via the thermal expansion method, using the adsorption of vermiculite and methylene blue and acid etching after high-temperature carbonization. On the basis of adsorbing various small molecule dyes, this is expected to be further economically and effectively used in the field of electrochemical energy storage. The construction of vermiculite hard template carbon/nitrogen–sulfur co-doped carbon nanosheet (CNS) composites provides a new strategy for the efficient application of natural vermiculite in the field of lithium-ion capacitors.

## 2. Results and Discussion

### 2.1. Microstructure and Composition

**Figure 1:**
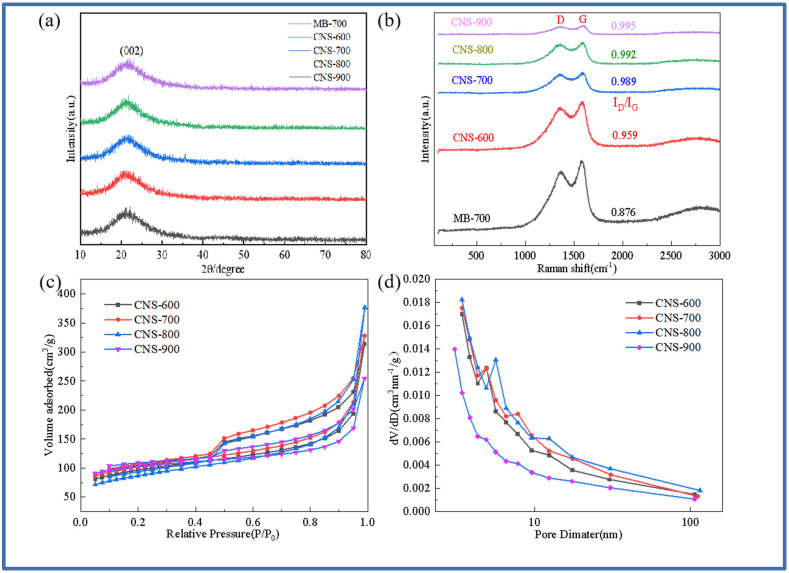
XRD patterns of MB-700, CNS-600, CNS-700, CNS-800, and CNS-900 (**a**); Raman spectra of MB-700, CNS-600, CNS-700, CNS-800, and CNS-900 (**b**); the nitrogen adsorption and desorption curves of vermiculite-based template carbon materials calcined at four different temperatures (**c**); and the pore size distribution curves of vermiculite-based template carbon materials calcined at four different temperatures (**d**).

Figure 1a,b show the XRD diffraction pattern and Raman spectrum of pure methylene blue carbon and vermiculite-based template carbon (MB-700, CNS-600, CNS-700, CNS-800, and CNS-900) prepared using methylene blue precursor at different temperatures. It can be seen from Figure 1a that a steamed bread-like diffraction peak appears near 23° in all five samples, indicating that the samples have an amorphous structure. There are no other diffraction peaks in the XRD patterns of these five carbon materials, indicating that the template carbon material templates treated at four different temperatures are basically eliminated. Figure 1b shows the Raman spectra of the five samples. At 1339 cm^−1^ and 1598 cm^−1^, a D peak representing the defects and amorphous structure of the carbon material and a G peak representing the carbon crystallite appear in the five materials. This shows that the methylene blue template carbon prepared at four different temperatures has a considerable microcrystalline structure and defect degree. The I_D_/I_G_ values of the five materials were 0.876, 0.959, 0.989, 0.992, and 0.995. From the I_D_/I_G_ ratio of the five materials, it can be seen that the graphitization of pure pyrolytic carbon without a template is lower than that of the template carbon materials. The ratio of template carbon materials prepared at four different temperatures increases with an increase in temperature. This is because the vermiculite template can hinder the graphitization of methylene blue [26]. In the interlayer domain of vermiculite, the confined space inhibits the activity of carbon, resulting in a decrease in the bonding probability of carbon atoms during graphitization.

Figure 1c,d show the nitrogen adsorption–desorption isotherms and pore size distribution curves of CNS-600, CNS-700, CNS-800, and CNS-900 prepared at different temperatures. The isotherms of the four template carbon materials show that, according to the IUPAC classification, the four samples all exhibit typical type IV isotherms with H3-type hysteresis loops, indicating that the stacking-free lamellar mesoporous structure of the nanoparticles is dominant [27]. It can be seen from the pore size distribution that the peak position has a slight shift. As the temperature increases, the size of the mesopores increases. In the relative pressure range of nitrogen (P/P_0_ < 0.1), the presence of micropores makes the adsorption amount increase sharply, and there is an obvious hysteresis loop in the medium relative pressure range (P/P_0_ = 0.4–0.9), which belongs to the presence of mesopores. In addition, the sharp increase in the isotherms at high relative pressure (P/P_0_ > 0.9) further indicates the existence of a macroporous structure. Macropores mainly come from the disordered accumulation of carbon nanosheets; micropores mainly come from the escape of small gas molecules during carbonization; and mesopores mainly come from the catalysis of metal elements on the surface of expanded vermiculite templates and the destruction of carbon nanosheets during template demolding. The porosity of the template carbon material can not only provide more active sites for the storage of lithium ions but also provide an effective way to transport lithium ions, thereby improving the specific capacity and rate performance of CNS as a lithium-ion battery anode. Table 1 shows the specific surface area and pore size data of the four samples. The pore structure of the template carbon prepared at four different temperatures is quite different from that of the pure carbon sample without a template. The specific surface areas of the four samples (CNS-600, CNS-700, CNS-800, and CNS-900) were 70.464 m^2^/g, 84.461 m^2^/g, 77.583 m^2^/g, and 76.332 m^2^/g, respectively. The average pore diameters were 3.409 nm, 3.408 nm, 3.410 nm, and 3.441 nm, respectively.

**Figure 2:**
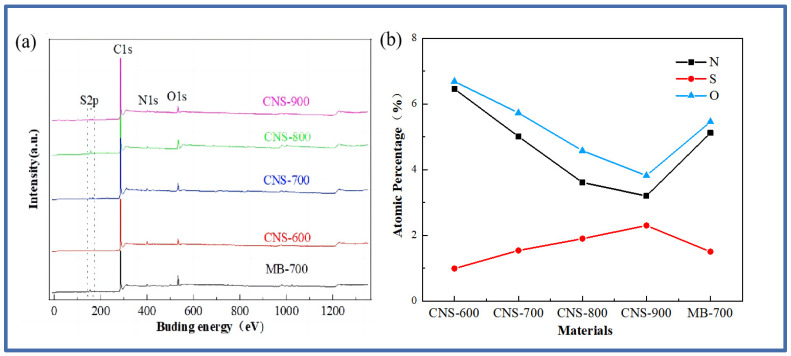
The XPS full spectrum (**a**) and the atomic percentage of N and S (**b**) in the five samples.

The surface chemical element composition of the five materials was analyzed, and the XPS test was carried out. Figure 2 is the XPS full spectrum of the five materials and the content of each element.

The full spectrum of Figure 2a shows that the five materials are mainly composed of the C, O, N, and S elements. The peak of S2p appears near 166 eV, the peak of C1s appears near 285 eV, the peak of N1s appears near 399 eV, and the peak of O1s appears near 532 eV. The appearance of the oxygen element is mainly derived from the oxygen element contained in the vermiculite. Specific information about the element content is shown in Figure 2b. It can be seen that the nitrogen content continues to decrease with the increase in temperature, while the sulfur element shows an increasing trend, which is also consistent with previous research [28].

**Figure 3:**
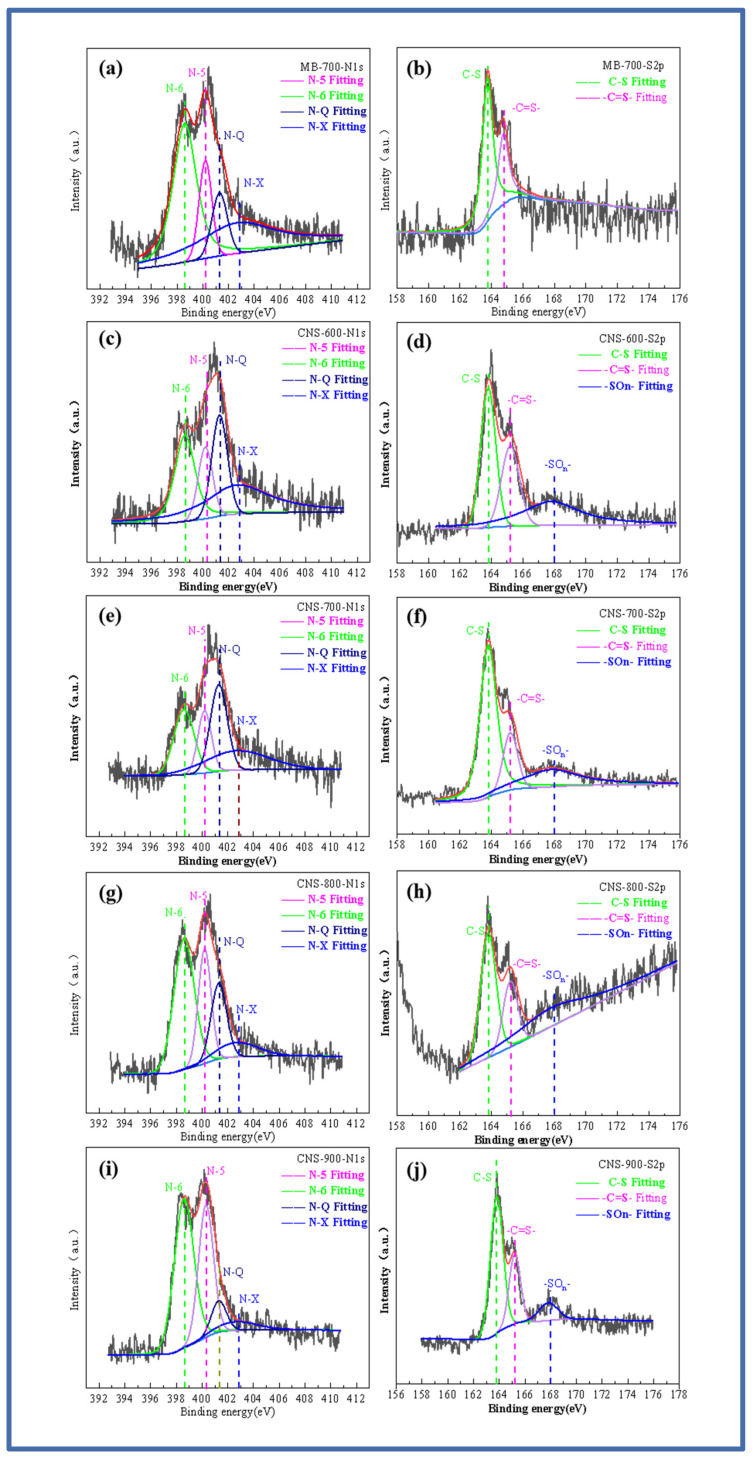
N1s and S2p fitting spectra of MB-700 (**a**,**b**), N1s and S2p fitting spectra of CNS-600 (**c**,**d**), N1s and S2p fitting spectra of CNS-700 (**e**,**f**), N1s and S2p fitting spectra of CNS-800 (**g**,**h**), N1s and S2p fitting spectra of CNS-900 (**i**,**j**).

In order to explore the doping state of the nitrogen and sulfur elements, Figure 3a–j show the high-resolution spectrum of the N and S elements. The high-resolution spectrum of N1s shows the binding energy of four peaks. They are located at 398.6 eV, 400.2 eV, 401.3 eV, and 402.7 eV, corresponding to pyridine nitrogen (N-6), pyrrole nitrogen (N-5), graphite nitrogen (N-Q), and pyridine nitrogen oxide (N-X) [29]. With the increase in temperature, the graphite nitrogen content shows an increasing trend, while the other three contents gradually decrease. This is mainly due to the higher thermal stability of graphite nitrogen, resulting in a relative increase in the percentage. After fitting the high-resolution spectrum of S2p, it can be seen that MB-700 pure pyrolytic carbon without a template, unlike the other four template carbon materials, can only fit two peaks of 163.3 and 164.4 eV, which is also consistent with previous studies on methylene blue. As the temperature increases, it can be seen that the S2p peaks of the template carbon materials prepared at four temperatures can be fitted into three peaks, and fitting peaks with binding energies of 163.3 and 164.4 eV correspond to the thiophene-structured sulfur (C-S), (-C=S-) S2p 1/2, and S2p 3/2. Thiophene-structured sulfur helps to improve the defect degree of the material itself and brings more active sites. In addition, the fitting peak, with a binding energy of 168 eV, corresponds to oxidized sulfur (-SOn-), which is mainly caused by the oxygen element between the vermiculite layers. With an increase in temperature, the oxidized sulfur (-SOn-) content also increases. The co-doped nitrogen and sulfur have an obvious synergistic effect, increasing the number of electrochemically active sites on the surface of the carbon materials and improving the charge distribution of active sites.

**Figure 4:**
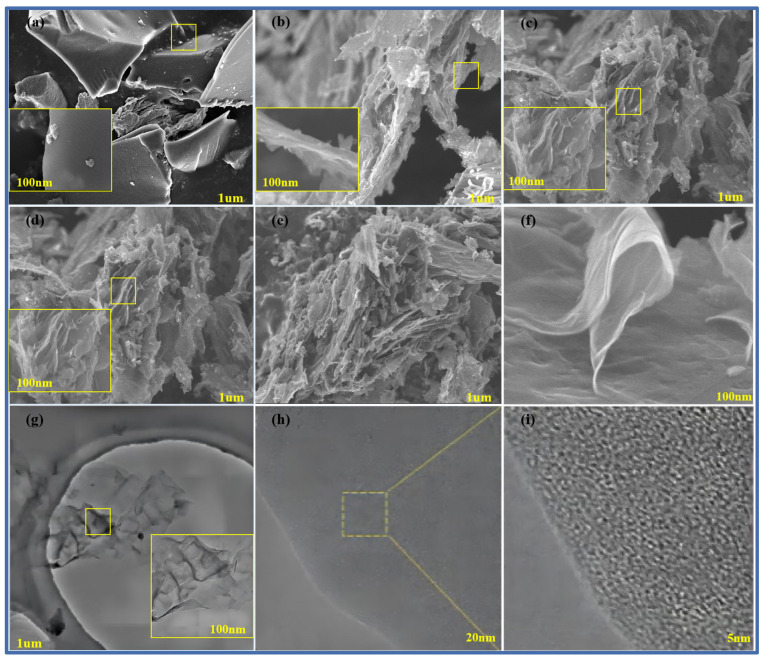
SEM map of MB-700 (**a**), CNS-600 (**b**), CNS-800 (**c**), CNS-900 (**d**), and CNS-700 for CNS (**e**,**f**); TEM image of CNS-700 (**g**); and HRTEM image of CNS-700 (**h**,**i**) (the small yellow frames in each graph refer to the locally enlarged regions).

Figure 4a–f show scanning electron microscope images of pure methylene blue carbon materials without a template and pure methylene blue carbon materials (MB-700, CNS-600, CNS-800, CNS-900, and CNS-700) without a template prepared at different temperatures. The small yellow frames in each graph refer to the locally enlarged regions. From the diagram, it can be seen that, compared with the block structure of the methylene blue carbon material control group, without adding the template, the carbon nanosheets prepared at other different temperatures all show an obvious irregular lamellar structure. It can be seen that the carbon nanosheets, after calcination and pickling, more completely inherit the layered structure of the vermiculite template (curly flake), and the surface is relatively smooth. This is also consistent with the results of the previous XRD analysis. In the four groups of carbon materials prepared at different temperatures, it can also be seen that, in the four groups of template carbon materials, the morphology is not much different.

In order to further observe the internal microstructure of CNS-700, it was analyzed with transmission electron microscopy, as shown in Figure 4g–i. Figure 4j shows a transmission electron microscope image of CNS-700, which is composed of various curly yarn structures, showing a typical graphene structure, and its morphology is also consistent with the scanning electron microscope image of CNS-700, measured above. Figure 4h shows that the irregular microcrystalline composition of the CNS-700 sample can be observed. In Figure 4i, the lattice spacing of CNS700 can be measured to be about 0.365 nm in high-resolution transmission electron microscope images.

### 2.2. Electrochemical Performance

**Figure 5:**
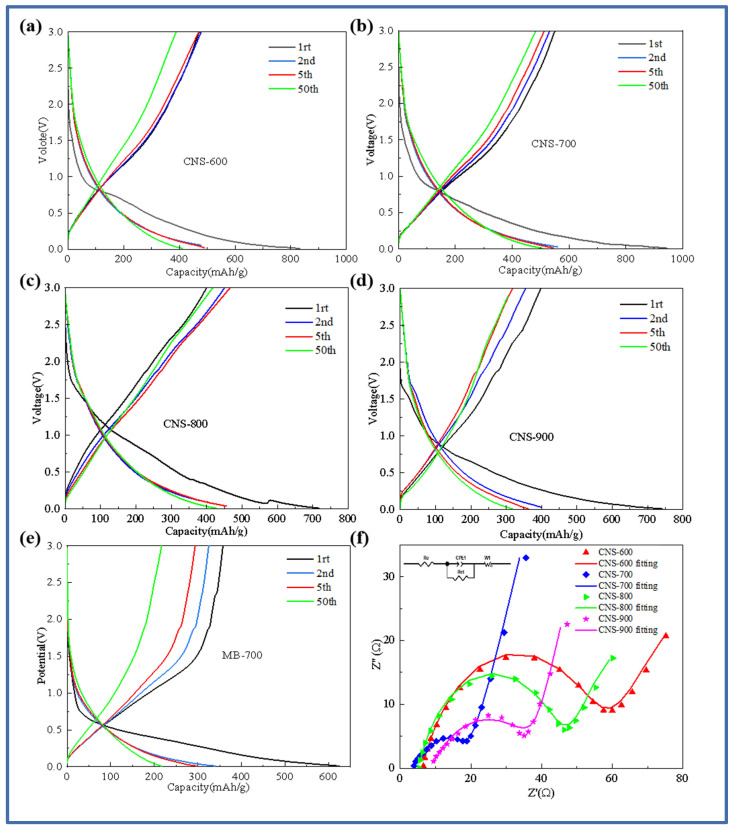
Charge–discharge curves of samples at different carbonization temperatures: CNS-600 (**a**), CNS-700 (**b**), CNS-800 (**c**), CNS-900 (**d**), MB-700 (**e**), and AC impedance spectra of samples at different carbonization temperatures (**f**).

In order to further study the electrochemical behavior of template carbon materials prepared at four temperatures and pure pyrolytic carbon without a template control structure, Figure 5 shows the charge–discharge curves of template carbon materials calcined at different temperatures and pure methylene blue pyrolytic carbon materials without a vermiculite template (CNS-600, CNS-700, CNS-800, CNS-900, and MB-700). Figure 5a–e show the first, second, fifth, and fiftieth charge–discharge curves of CNS-600, CNS-700, CNS-800, CNS-900, and MB-700 at a current density of 0.1 A/g. As shown in Figure 5a–e, in the first charge and discharge cycle, the samples at different temperatures showed an obvious voltage platform near 0.75 V, corresponding to the decomposition of lithium-containing electrolytes and the formation of SEI (solid electrolyte interface membrane). The results also correspond to the CV curve in the following figure. CNS-600, CNS-700, CNS-800, CNS-900, and MB-700 showed specific capacities of 839.2 mAh/g, 1100.3 mAh/g, 717.4 mAh/g, 742.1 mAh/g, and 624.4 mAh/g in the first discharge and 513 mAh/g, 649.8 mAh/g, 404.1 mAh/g, 399.6 mAh/g, and 357.7 mAh/g in the first charge, respectively. The first coulombic efficiencies were 63.6%, 64.7%, 62.1%, 53.8%, and 57.2%, respectively.

The first discharge-specific capacity and the first coulombic efficiency of the nitrogen–sulfur co-doped template carbon material (CNS-700) with a carbonization temperature of 700 °C are the highest among the three. A large number of regular and ordered graphite-like layer structures provide abundant reversible intercalation space for lithium ions. The large specific surface area and well-developed mesoporous structure provide active sites for Li^+^ storage [30]. A large number of regular and ordered graphite-like layer structures improve the conductivity of the anode material, and a small number of micropore structure defects effectively reduce the reversible capacity loss caused by the irreversible desorption of lithium ions. These two structural features together achieve high first-Coulomb efficiency. At the same time, the curve changes of the four template carbon materials after the second charge–discharge cycle begin to decrease significantly, indicating that the template carbon materials prepared at three temperatures have good stability. After 50 cycles, the specific capacity can be stabilized at about 450 mAh/g, and the efficiency is almost 100%, showing the excellent cycle stability of the template carbon material during charge and discharge.

An electrochemical impedance spectroscopy (EIS) analysis reveals the dynamic process of the battery operation process of the four samples. Figure 5f is a Nyquist diagram of the five samples and the fitting circuit.

From Figure 5f, it can be seen that the impedance spectra of the four samples are composed of two regions: a semicircle in the high-frequency region, representing the resistance, RSEI, caused by the SEI film, and the charge transfer resistance, Rct [31]. The diagonal in the low-frequency region belongs to Warburg impedance related to the lithium-ion diffusion process; the template carbon samples prepared at four different temperatures generally show a trend of decreasing first and then increasing with an increase in temperature. The resistance values of the fitting circuit components of the four template carbon materials are shown in Table 2.

CNS-700 has the smallest solution resistance (5.35 Ω) and charge transfer resistance (19.05 Ω). This is mainly because the morphology of vermiculite can be better maintained at 700 °C, and the resulting multilayer graphene-like structure and appropriate heteroatom doping provide more defects for the material, which is conducive to the conductivity of lithium-ion diffusion.

**Figure 6:**
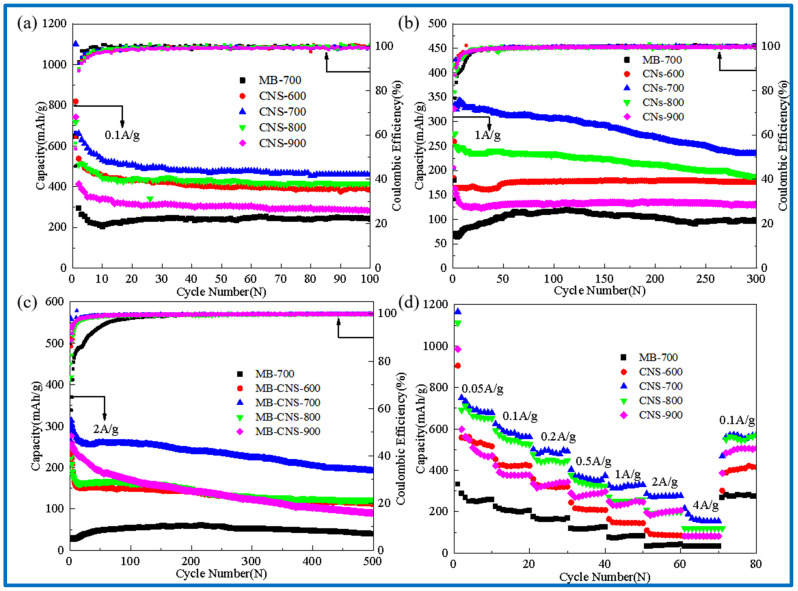
Cycle performance diagram at 0.1 A/g (**a**); cycle performance diagram at 1 A/g (**b**); cycle performance diagram at 2 A/g (**c**); rate performance diagrams of 0.05, 0.1, 0.2, 0.5, 1, 2, 4 A/g (**d**).

Figure 6a–d show cyclic performance diagrams of the template carbon and the blank carbon materials without a template at different carbonization temperatures (a) at a current density of 0.1 A/g, (b) at a current density of 1 A/g, (c) at a current density of 2 A/g, and (d) at different current densities.

In Figure 6a, the discharge-specific capacities of CNS-600, 700, 800, 900, and MB-700 after 100 cycles at a current density of 0.1 A/g are 383, 460, 415, 282, and 243 mAh/g, respectively. The capacity retention rates are much higher than those of the methylene blue pyrolytic carbon (MB-700) without adding templates, indicating that the carbon precursors of the four template carbon materials at different temperatures form intercalated two-dimensional carbon nanosheets with vermiculite, which is also consistent with the results in the previous scanning electron microscopy.

It can be seen that CNS-700 exhibits the best cycle stability. The reversible capacity can still reach 460 mAh/g after 100 cycles at a current density of 0.1 A/g. The reversible specific capacity can reach 300 mAh/g after 300 cycles at a current density of 1 A/g. The reversible specific capacity can still reach 200 mAh/g after 500 cycles at 2 A/g, showing excellent rate performance. This may be due to the relatively large specific surface area, a small number of micropores, and the developed mesoporous structure of CNS-700, and a more ordered and stable graphite-like microcrystalline layer structure is produced at a carbonization temperature of 700 °C.

**Figure 7:**
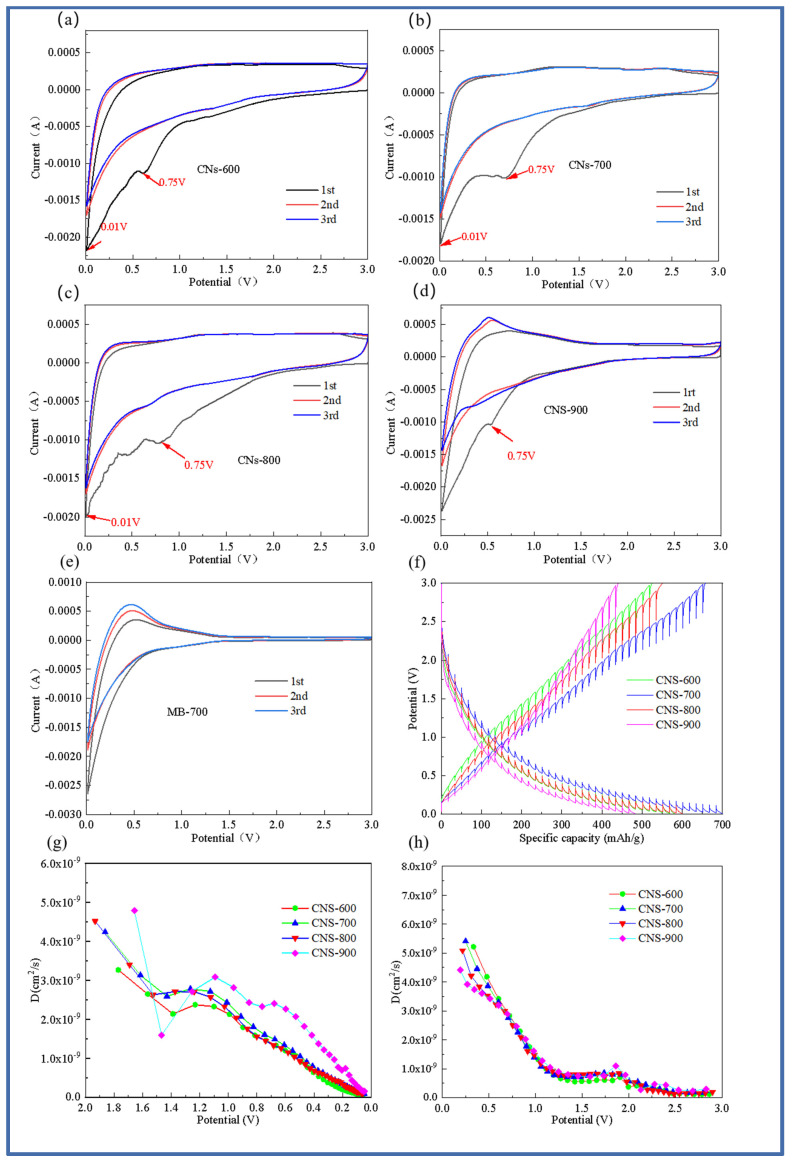
Cyclic voltammograms of four templated carbon materials. CNS-600 (**a**), CNS-700 (**b**), CNS-800 (**c**), CNS-900 (**d**), and carbon material MB-700 prepared at different calcination temperatures (**e**); GITT test curve of four template carbon materials in the charging and discharging process (**f**); lithium-ion diffusion coefficient of four template carbon materials in the discharging process (**g**); lithium-ion diffusion coefficient curve of four template carbon materials in the charging process (**h**).

The cyclic voltammetry curves of the first, second, and third cycles of the five samples (CNS-600, CNS-700, CNS-800, CNS-900, and MB-700) at 0.5 mV/s are shown in Figure 7. Compared with the MB-700 sample without a template for morphology control, the four template carbon samples all showed typical CV curves for graphene materials [32]. The cycle curve of MB-700, the product of the direct pyrolysis of methylene blue, shows the characteristics of ordinary hard carbon, which also indicates that the prepared template carbon material is a graphene-like carbon sheet. In the first discharge cycle, there are obvious reduction peaks near 0.75 and 0.01 V. The reduction peak near 0.75 V indicates the formation of SEI film [33]. During the charging process, the oxidation peak near 0.3 V mainly corresponds to the chemical process of lithium electrolyte oxidation into lithium ions. In addition, it can be seen from the figure that, compared with the pure methylene blue carbon source control group without a vermiculite template, the curve area of the template prepared at four different temperatures is larger than that of pure carbon, and the curve overlap is higher, indicating that the template carbon material can effectively improve the reversible capacity, which is also consistent with the previous constant current charge and discharge results. It can be observed from the CV curves of the template carbon materials prepared at four different temperatures that CNS-700 has the best coincidence degree, indicating that a relatively stable SEI film is formed on its surface and that it has good cycle stability.

In order to analyze the lithium-ion diffusion phenomenon of the material at various voltages, the four template carbon materials were tested with GITT (Gradient Impedance Trap Technology), as shown in Figure 7f–h. Data from 10 cycles at a current density of 0.1 A/g were selected for analysis to ensure the stability and reliability of the data. In order to further explore the diffusion kinetics of four template carbon electrode materials, Figure 7f represents the GITT curve of the five electrode materials during charge and discharge, and the lithium-ion diffusion coefficient in the working voltage serial port is calculated based on Fick’s second law. Figure 7g is the diffusion coefficient curve of the five electrode materials during the discharge process. It can be seen that, in a voltage range of 2.0~1.4 V, the lithium-ion diffusion coefficient of the five electrode materials decreases rapidly with the discharge process, which is mainly because of the continuous insertion of lithium ions.

In the voltage range of 1.4 V–1.2 V, the diffusion coefficient begins to maintain within a certain range, indicating that this stage is at the equilibrium point of lithium ion insertion and extraction. In the range of 1.2 V–0.1 V, the diffusion coefficient is in a continuous downward trend. Similarly, the four template carbon materials also showed the same trend during the charging process, and the diffusion coefficient was on the order of 10^−9^ cm^2^s^−1^.

From the lithium-ion diffusion coefficient curves of the four template carbon materials in Figure 6h, it is easy to see that the lithium-ion diffusion coefficient in the low potential platform region is also relatively low. In addition, it can be seen that the lithium-ion diffusion coefficient of the CNS-700 sample always maintains a leading position during the charge and discharge process, which is also consistent with the AC impedance results.

**Figure 8:**
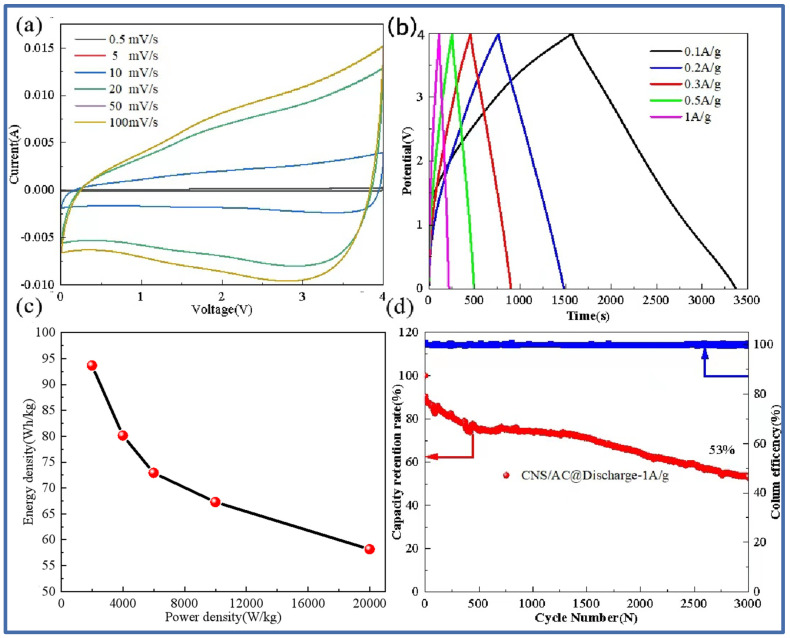
CNS-700//AC lithium-ion capacitor cyclic volt–ampere curve at different scanning rates (**a**); constant current charge–discharge curve at different current densities (**b**); Thouragongtu (**c**); long cycle capacity retention rate and coulomb efficiency diagram (**d**).

Figure 8 shows the cyclic voltammetry curve at different scanning rates, the charge–discharge curve at different current densities, the energy density and power density curve, and the long cycle capacity retention curve of the lithium-ion capacitor assembled by the negative CNS-700 electrode and the positive AC electrode. Figure 8a shows the cyclic voltammetry curves of the CNS-700//AC lithium-ion capacitors at different scan rates (0.5 mV/s–100 mV/s). It can be seen that, when the scanning rate is 0.5 mV/s, the CV curve shows a rectangular shape, which shows that the energy storage of the lithium-ion capacitor not only has the capacitance behavior of the capacitor but also contains the redox reaction process of the lithium battery. At the same time, it can also be observed that, as the scanning rate continues to increase, the scanning curves basically maintain good rectangular characteristics, indicating that the mass matching of the positive and negative electrodes is good. Figure 8b is the time–voltage curve at different current densities (0.1 A/g–1 A/g). The results show that the time–voltage curve is symmetrical and triangular, indicating that CNS-700//AC has good reversibility and shows excellent capacitance performance. The relationship between the energy density and the power density of the device can be calculated, as shown in Figure 8c. Specifically, the energy density of the CNS-700//AC lithium-ion capacitor is 93.67 Wh/kg at a power density of 2000 W/kg; the energy density is 58.17 Wh/kg at a power density of 20 kW/kg. As shown in Figure 8d, the lithium-ion capacitor has a capacity retention rate of 53% after 3000 cycles at a current density of 1 A/g, showing the excellent cycle stability of the full battery.

## 3. Materials and Methods

### 3.1. Preparation of Materials

The vermiculite (VMT) was from Yuli, Xinjiang, and the reagents were purchased from the State Pharmaceutical Company. The experimental steps for preparing CNS composites are as follows.

1. The vermiculite raw mineral water was cleaned and dried after filtering impurities such as soil and sand. Subsequently, the dried vermiculite was calcined in a muffle furnace at 1000 °C for 1 min, and the thermally expanded vermiculite obtained via sieving was recorded as EV.
2. In total, 10 g of methylene blue (C_16_H_18_ClN_3_S·3H_2_O) was dispersed in 100 mL of deionized water. Subsequently, the expanded vermiculite was added to the mixed solution. After ultrasonic treatment for 3 h and vacuum immersion for 12 h, the methylene blue molecules were fully adsorbed to the surface or entered the gap of the expanded vermiculite layer. After filtration and drying, the intercalation compound was recorded as MB-EVMT.
3. The intercalation compounds were carbonized at 600 °C, 700 °C, 800 °C, and 900 °C for 2 h in N_2_ atmosphere and cooled to room temperature. The product prepared via carbonization at 600 °C was labeled as MB-CNS-600, and the products prepared at different temperatures were labeled with the same method.
4. MB-CNS-600, MB-CNS-700, MB-CNS-800, and MB-CNS-900 were soaked in hydrofluoric acid (HF) and concentrated hydrochloric acid (HCl) to remove the template, washed with deionized water to neutral, suction filtrated, and dried in a drying cabinet. Finally, we obtained CNS-600, CNS-700, CNS-800, and CNS-900.
5. Pure methylene blue without a vermiculite template was carbonized at 700 °C for 2 h in a N_2_ atmosphere. The product obtained via cooling and sieving was marked as MB-700.

### 3.2. Materials Characterization

The structure of the samples was characterized by XRD-6100, Cu Kα, a tube voltage of 40 V, a tube current of 40 mA, a scanning angle of 5°–80°, a step length of 0.04°, and a scanning speed of 10°/min. Microstructures were investigated using a scanning electron microscope (SEM) (JSM-7500F, JEOL, Tokyo, Japan). A micro-Raman spectrometer (InVia RM 1000, Renishaw), produced in London, UK, was used to test the microstructure characterization of the prepared materials. The wavelength was selected as 532 nm, and the scanning range was 200–3000 cm^−1^. A Japanese company’s JEM-2100F (JEOL, Tokyo, Japan) was used to further observe the surface and internal microstructure of the material, and the device was used to observe the lattice fringes under a high-resolution transmission electron microscope to verify the crystal structure. In addition, at high multiples, the lattice fringes were observed to verify the crystal structure. The specific surface area and pore structure of the material were measured with an Autosorb-IQ type tester. The adsorption–desorption curve of the material was obtained by drawing the relationship curve between nitrogen adsorption and pressure.

### 3.3. Electrochemical Measurements

The CNS material, acetylene black, and binder (PVDF) obtained at different carbonization temperatures were mixed in N-methyl pyrrolidone (NMP) solvent at a mass ratio of 8:1:1 to form a uniform slurry. It was then coated on a copper foil for the anode. The copper foil was dried in a vacuum oven and then pressed into a negative pole piece. Activated carbon (AC) was used as the active material of the positive electrode. The preparation method for the positive electrode was the same as that of the negative electrode, and the AC active material was coated on the aluminum foil with the same method. In total, 1 mol/L LiPF_6_ of electrolyte (solvent is dimethyl carbonate (DMC)/methyl ethyl carbonate (EMC)/ethylene carbonate (EC); volume ratio, 1:1:1) and a separator were placed between the counter electrode (AC) and the working negative electrode, respectively. Finally, a sealing machine was used in the glove box to complete the packaging of the capacitor.

Given the above steps, the CNS material obtained at different carbonization temperatures was used as the negative electrode active material, and a lithium metal sheet was used to replace the active carbon (AC) of the capacitor’s positive electrode active material. A half-cell was assembled.

Constant current charge and discharge tests were performed using a BTS-5 V/2.2 A battery tester, and rate performance tests were performed at different current densities. The half-cell test voltage range was 0.01~3.0 V, and the lithium-ion capacitor test voltage range was 0.01~4.0 V. Electrochemical impedance spectroscopy (EIS, 0.01–10^5^ Hz) and cyclic voltammetry (CV) tests were performed using an electrochemical workstation (CHI660E, CHI-Instrument). The GITT test data were obtained from a BTS-5 V/2.2A tester, and the test data were obtained at room temperature and after cycle stability.

## 4. Conclusions

Nitrogen and sulfur co-doped two-dimensional carbon nanosheets were prepared by using expanded vermiculite as a template and methylene blue as a carbon source to form a confined space between vermiculite layers. The effects of temperature on the internal structure and the electrochemical properties of heteroatom-doped carbon were studied with phase and electrochemical tests. The carbonization temperature affected the formation of carbon nanosheets and the content of heteroatoms in situ. The results show that the surface smoothness and microstructure of this template carbon material can be affected by adjusting the carbonization temperature. When the temperature is 700 °C, and the carbonization time is 2 h, the current density is 0.1 A/g, the first discharge capacity can reach 850 mAh/g, and the first coulombic efficiency can reach 64.7%. After 100 cycles of charge and discharge, the discharge-specific capacity can still reach 450 mAh/g. Moreover, the material has good rate performance and can retain a specific capacity of 200 mAh/g after 500 cycles at a current density of 2 A/g. The lithium-ion capacitor was further assembled by using CNS-700 as the negative electrode and commercial activated carbon (AC) as the positive electrode. Table 3 integrates the energy density/power density data concerning the LICs in some of the related literature and compares them with the LICs assembled in this experiment. The electrochemical test showed excellent electrochemical performance. The assembled capacitor had excellent cycle stability (the 3000-cycle capacity retention rate was 53%), and the energy density was 93.67 Wh/kg at a power density of 2000 W/kg. The energy density was 58.17 Wh/kg at a power density of 20,000 W/kg, and the electrochemical performance was excellent.

## Figures and Tables

**Table 1:** Specific surface area and aperture data.

Sample Name	Specific Surface Area (m^2^/g)	Hole Volume (cm^3^/g)	Average Pore Size (nm)
CNS-600	70.464	0.352	3.409
CNS-700	84.461	0.369	3.408
CNS-800	77.583	0.469	3.410
CNS-900	76.332	0.483	3.441

**Table 2:** Fitting values of CNs-600, 700, 800, and 900 impedance fitting circuit elements.

Sample Name	Fitted Value			
	R_S_/Ω	R_ct_/Ω	CPE/Ω^−1^·cm^−2^·s^−n^	W_0_/Ω
CNS-600	5.49	33.18	6.22 × 10^−5^	3.57
CNS-700	5.35	19.05	6.22 × 10^−5^	3.57
CNS-800	8.30	43.66	6.22 × 10^−5^	3.57
CNS-900	8.67	27.78	6.22 × 10^−5^	3.57

**Table 3:** Electrochemical performance of capacitors in different systems.

System (Anode//Cathode)	Energy Density (Wh/kg)	Power Density (KW/kg)	References
CNS-700//AC	58.17	20.0	This work
GDPC-1//AC	50.50	20.0	[34]
MnO/NCN//AC	53.00	6.8	[35]
HNBC//AC	78.00	11.0	[36]
BNC//AC	53.60	10.0	[37]
PHC-4//AC	41.00	12.0	[38]

## Data Availability

Data are contained within the article.
